# Cell Toxicity Study of Antiseptic Solutions Containing Povidone–Iodine and Hydrogen Peroxide

**DOI:** 10.3390/diagnostics12082021

**Published:** 2022-08-21

**Authors:** Veronica Romano, Donato Di Gennaro, Anna Maria Sacco, Enrico Festa, Emanuela Roscetto, Morena Anna Basso, Tiziana Ascione, Giovanni Balato

**Affiliations:** 1 Section of Human Anatomy, Department of Public Health, “Federico II” University, 80131 Naples, Italy; 2 Section of Orthopaedic Surgery, Department of Public Health, “Federico II” University, 80131 Naples, Italy; 3 Section of Clinical Microbiology, Department Molecular Medicine and Medical Biotechnology, “Federico II” University, 80131 Naples, Italy; 4 Department of Medicine, Service of Infectious Disease, Cardarelli Hospital Naples, 80131 Naples, Italy

**Keywords:** antiseptics, povidone–iodine, hydrogen peroxide, infection

## Abstract

The increasing incidence of periprosthetic joint infections (PJIs) has led to a growing interest in developing strategies to prevent and treat this severe complication. The surgical site’s application of antiseptic solutions to eliminate contaminating bacteria and eradicate the bacterial biofilm has been increasing over time. Even though it has been proven that combining antimicrobials could enhance their activities and help overcome acquired microbial resistance related to the topical use of antibiotics, the toxicity of integrated solutions is not well described. This study aimed to evaluate the cytotoxicity of solutions containing povidone–iodine (PI) and hydrogen peroxide (H${}_{2}$O${}_{2}$), alone or in combination, after 1.3 and 5 min of exposure. Chondrocytes, tenocytes, and fibroblast-like synoviocytes were used for cytotoxicity analysis. Trypan blue stain (0.4% in PBS) was applied to evaluate the dead cells. All solutions tested showed a progressive increase in toxicity as exposure time increased except for PI at 0.3%, which exhibited the lowest toxicity. The combined solutions reported a reduced cellular killing at 3 and 5 min than H${}_{2}$O${}_{2}$ at equal concentrations, similar results to PI solutions.

## 1. Introduction

Periprosthetic joint infections (PJIs) are one of the most severe complications in modern orthopedics. Although the incidence of PJIs, for hip and knee ranges between 1 and 2 percent, the number of postoperative infections has been estimated to increase with substantial economic and social repercussions [1]. The severity of these medical conditions is related to the ability of the contaminating bacteria to produce biofilm, a community of micro-organisms irreversibly attached to a biological or inert surface and encased in a slime produced by the micro-organisms themselves. The biofilm formation may lead to increased resistance to host responses and antibiotics, thus causing chronic infections whose treatment is still highly demanding. The treatment of PJI depends on the type of infection (acute vs. chronic), the causative microorganism, and the host bone and soft tissues. In particular, the treatment consists of the mechanical removal of all infected tissues (debridement) and contaminated implant and the revision of prosthesis in one or two surgical stages associated with long-lasting antibiotic therapy. Two-stage revision arthroplasty is a well-established method of care for patients with chronic PJIs and provides the use of a cement spacer and, subsequently, revision prosthesis once the infection is considered eradicated [2,3,4]. For these reasons, it is essential to find a solution that is capable of eliminating contaminating bacteria and eradicating the bacterial biofilm, thus reducing the incidence rate of post-operative infections and increasing the success rate of the treatment options. A possibility to reduce the bacterial load and eradicate the biofilm is the use of silver-coated implants or the use of antiseptic agents at the surgical site [5]. Different solutions using various antiseptics alone or in association are described. The most widely used antiseptic is diluted povidone–iodine (PI), which, thanks to its safety profile, effectiveness, and low cost, is currently the only recommended surgical site irrigation [2,3,6,7,8,9]. Similarly, hydrogen peroxide (H${}_{2}$O${}_{2}$) is the other most widely used antiseptic, often associated with iodopovidone, especially in spine surgery but also in case of arthroplasty [10,11]. Recently, a new irrigation solution which consists of 500 mL of 0.9 percent saline sterile solutionwith 18 mL of 10 percent aqueous PI and 125 mL of 3 percent H${}_{2}$O${}_{2}$ added was proposed as an adjuvant in the treatment of PJI [12]. The rationale for using PI and H${}_{2}$O${}_{2}$ together is to obtain a synergistic action of the two antiseptics, thus generating a bactericidal effect [3]. Even though it has been proven that combining antimicrobials could enhance their activities and help overcome acquired microbial resistance related to the topical use of antibiotics, the toxicity of integrated solutions is not well described. This study aims to evaluate the cytotoxicity of the solution of PI and H${}_{2}$O${}_{2}$ alone or in combination. The proposed solutions are tested on chondrocytes, tenocytes, and fibroblast-like synoviocytes cultures.

## 2. Materials and Methods

### 2.1. Antiseptics Solutions

All solutions, as shown in Table 1, were prepared to start from a solution of 10% povidone–iodine and 3% H${}_{2}$O${}_{2}$. We proceeded to the dilution with NaCl 0.9% and then to the combination to obtain the final solutions. The solutions were prepared under sterile conditions and filtered using a 0.2 μm filter.

### 2.2. Cell Culture

The Human Fibroblast-Like Synoviocytes (HFLS), Normal Human knee Articular Chondrocytes (NHAC-kn), and Human Tenocytes were purchased from Sigma-Aldrich (St. Louis, MO, USA), Lonza (Walkersville, MD, USA) and AcceGen (Fairfield, NJ, USA). Cells were plated in 35 mm dishes and cultured in DMEM High Glucose (Sigma-Aldrich) enriched with 10% fetal bovine serum (Sigma-Aldrich), penicillin 10,000 U, and streptomycin 10 mg/mL (Sigma-Aldrich), at 37 ${}^{\circ }$C in 5% CO${}_{2}$. Plates were observed daily at an inverted phase-contrast microscope (Olympus, Segrate, Italy), and the medium was replaced every three days. Once the adherent cells were more than 75% confluent, they were detached with 0.25% trypsin-EDTA (Sigma-Aldrich) and then expanded. For the analysis, 15 × 104 cells were seeded in 35 mm dishes at 37 ${}^{\circ }$C in 5% CO${}_{2}$. The medium was aspirated, and cells were exposed to 1 mL of various irrigation solutions for 1, 3, and 5 min.

### 2.3. Cytotoxicity Analysis

After the exposition of the different solutions, cells were detached from the plates by incubation with 0.25% trypsin-EDTA solution (Sigma-Aldrich) for 10 min. Detached cells were then stained with trypan blue stain (0.4% in PBS) (Lonza) for 2 min at room temperature and counted by the observer using a hemocytometer. Dead cells uptake the dye due to damage to the plasma membrane, thus staining themselves blue. Live cells, instead, exclude the dye and can be recognized among the dead blue cells as unstained cells. All solutions have been tested for each cell line for all exposure times for three replicates.

### 2.4. Statistical Analysis

The percentage of dead cells over total cells for each exposure time was calculated and expressed as the mean percentages ± SD. Data were analyzed using a one-way analysis of variance, followed by Tukey’s post hoc test. This test was used to compare (i) the mean of a single solution at a different time of exposure (1.3 and 5 min) and (ii) the effects of the cellular killing of different solutions at the same time of exposure. Furthermore, for statistically significant differences, we performed a post hoc test as a subgroup analysis to highlight the differences between groups. The level of significance was set at *p* < 0.05. The IBM SPSS Statistics for Windows, Version 23.0 (IBM Corp., Armonk, NY, USA) was used for database construction and statistical analysis.

## 3. Results

### 3.1. Chondrocytes

**Figure 1:**
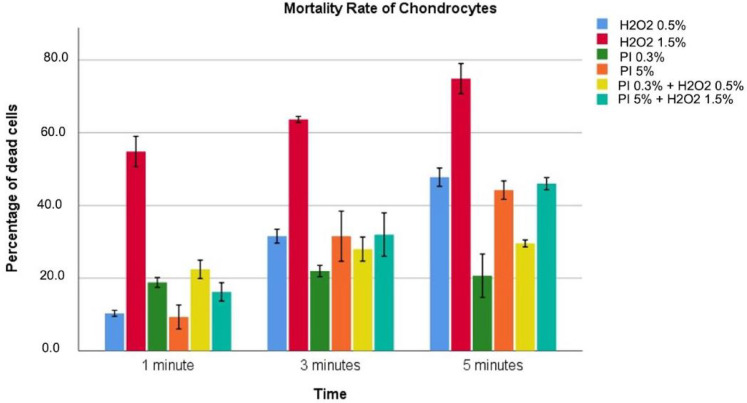
Different colors indicate different solutions. The height indicates the percentage of dead cells for the highlighted solution at that specific exposure time associated with 95%CI.

Data regarding cytotoxicity for chondrocytes are reported in Table 2 and graphically described in Figure 1.

Hydrogen peroxide solutions, combined solutions, and PI at 5% showed a progressive increase in toxicity as exposure time increased. PI 0.3% showed this increase only at 1 and 3 min without further increments at 5 min. H${}_{2}$O${}_{2}$ solutions also showed a concentration dependence with toxicity significantly greater for 1.5% compared to 0.5% independently from the exposure time. PI solutions, instead, appear to have toxicity not directly related to the concentration, with PI at 0.3% more toxic than PI at 5%, for an exposure time of 1 min, with no statistical significance (18.8 ± 0.53 vs. 9.33 ± 1.2, *p* = 0.118). Concerning the combined solutions, the solution (PI 0.3% + H${}_{2}$O${}_{2}$ 0.5%) showed more significant toxicity at 1 min but reported a reduced cellular killing at 3 and 5 min compared to PI 5% + H${}_{2}$O${}_{2}$ 1.5% solution. Furthermore, PI 0.3% + H${}_{2}$O${}_{2}$ 0.5% solution at 3 and 5 min of exposure showed a lower toxicity compared to all the evaluated solutions except for PI solution at 0.3%. We found that the H${}_{2}$O${}_{2}$ 1.5% solution was the most toxic for all exposure times considered, with maximum toxicity reported after 5 min of exposure. In contrast, dead cells of 21.9% and 20.7%, respectively.

### 3.2. Tenocytes

**Figure 2:**
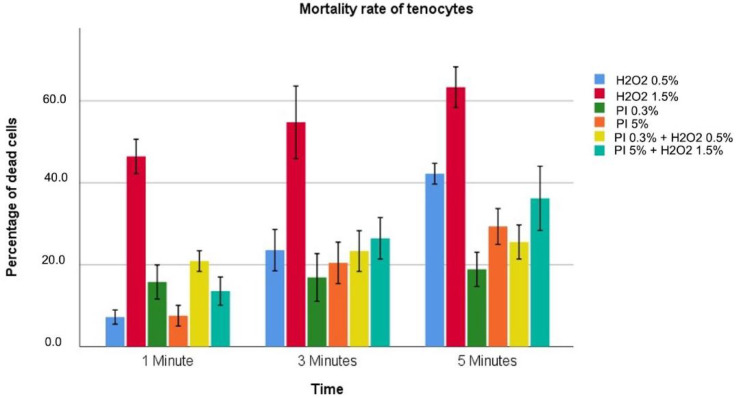
Different colors indicate different solutions. The height indicates the percentage of dead cells for the highlighted solution at that specific exposure time associated with 95%CI.

Data regarding cytotoxicity for tenocytes are reported in Table 3 and graphically described in Figure 2.

All solutions tested showed a progressive increase in toxicity as exposure time increased. This increase was reported as not statistically significant for PI at 0.3% (*p* = 0.214). H${}_{2}$O${}_{2}$ solutions also showed a concentration dependence with toxicity significantly greater for 1.5% compared to 0.5%. PI solutions instead appear to have toxicity not directly related to the concentration, with PI at 0.3% more toxic for an exposure time of 1 min than PI at 5% (15.8 ± 1.7 vs. 7.6 ± 1.0; *p* < 0.001). Concerning the combined solutions, the solution (PI 0.3% + H${}_{2}$O${}_{2}$ 0.5%) showed more significant toxicity at 1 min but reported a reduced cellular killing at 3 and 5 min compared to PI 5% + H${}_{2}$O${}_{2}$ 1.5% solution. H${}_{2}$O${}_{2}$ at 1.5% is the most toxic for all exposure times considered, with maximum toxicity of 63.3% at 5 min. H${}_{2}$O${}_{2}$ 0.5% and PI 0.3% were the less toxic solutions after 1 min and 3 and 5 min of exposure, respectively.

### 3.3. Fibroblast-like Synoviocytes

**Figure 3:**
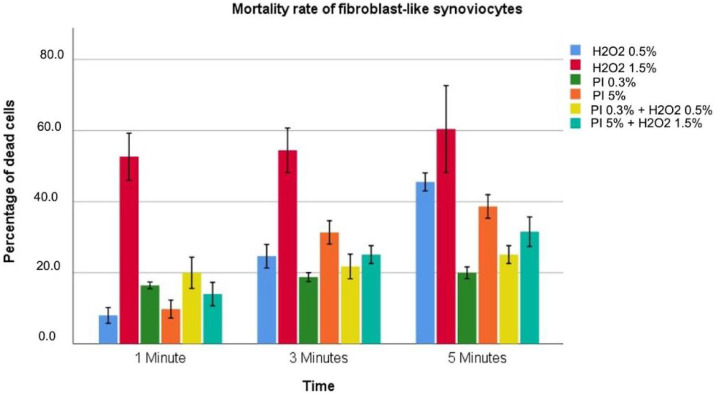
Different colors indicate different solutions. The height indicates the percentage of dead cells for the highlighted solution at that specific exposure time associated with 95%CI.

Data regarding cytotoxicity for fibroblast-like synoviocytes are reported in Table 4 and graphically described in Figure 3.

All solutions tested showed a progressive increase in toxicity as exposure time increased. H${}_{2}$O${}_{2}$ solutions also showed a concentration dependence with toxicity significantly greater for 1.5% compared to 0.5% (*p* < 0.001). PI solutions instead appear to have toxicity not directly related to the concentration, with PI at 0.3% more toxic for an exposure time of 1 min than PI at 5% (16.4 ± 0.3 vs. 9.8 ± 1.0; *p* < 0.001). The combined solutions PI 0.3% + H${}_{2}$O${}_{2}$ 0.5% showed a greater toxicity at 1 min (20.0 ± 1.8 vs. 14.0 ± 1.3; *p* < 0.001). In contrast, no statistically significant difference was retrieved after 3 min of exposure (21.8 ± 1.4 vs. 25.1 ± 1.0; *p* = 0.134), while at 5 min of exposure a higher toxicity rate for povidone–iodine 5%/hydrogen peroxide 1.5% was observed (31.6 ± 1.7 vs. 25.1 ± 1.0, *p* < 0.001). H${}_{2}$O${}_{2}$ 1.5% was found to be the most toxic for all exposure times considered, with a maximum toxicity of 60.4% at 5 min. H${}_{2}$O${}_{2}$ 0.5% solution was the least toxic for 1 min with 8% and PI 0.3% for 3 and 5 min with 18.9% and 20%, respectively. Finally, PI 0.3% + H${}_{2}$O${}_{2}$ 0.5% showed compared results of PI 0.3% after 3 and 5 min of exposure (21.8 ± 1.4 vs. 18.9 ± 0.5, *p* = 0.204; 25.1 ± 1.0 vs. 20.0 ± 0.7, *p* = 0.137).

## 4. Discussions

The ideal antiseptic solution for intraoperative use remains unknown. This study aimed to evaluate the cytotoxicity of different solutions containing povidone–iodine and hydrogen peroxide alone or in an association. To the best of our knowledge, this is the first study that considers different combinations of povidone–iodine and hydrogen peroxide and evaluates the cytotoxicity on tenocytes, as well as chondrocytes and fibroblast-like synoviocyte cells. Although previous studies have investigated the cytotoxic effects of antiseptic compounds, they often consider longer exposure times, which are more relevant for other applications such as managing surgical wounds. Instead, this study highlighted the different behavior of the antiseptic solutions considering the exposure time and the cellular line. The H${}_{2}$O${}_{2}$ solutions showed time and concentration-dependent toxicity. Indeed, H${}_{2}$O${}_{2}$ at 1.5% was proved to be the most toxic solution for all exposure times and all cell lines, especially for chondrocytes. In contrast, H${}_{2}$O${}_{2}$ at 0.5% proved to be among the least toxic if used for 1 min, but with a marked increase in toxicity at 3 and 5 min. These results align with Schaumburger et al. [13], who found cellular mortality of close to 75% for chondrocytes when exposed to H${}_{2}$O${}_{2}$ at 1% for 5 min. However, this study evaluated the cytotoxicity after 20 h from exposure in contrast to our paper in which the mortality rate was tested immediately after exposure. In addition, Lineaweaver et al. [14] found a very high mortality rate for fibroblasts when exposed to solutions ranging from 0.3% to 3% of H${}_{2}$O${}_{2}$. Although H${}_{2}$O${}_{2}$ at 1.5% was proved to be the most toxic solution for fibroblast cells, we did not find such high mortality rates. This reflects cytotoxicity’s strong exposure time dependence, considering the higher exposure times reported by Lineaweaver et al. [14] compared to ours (5 min vs. 15 min). PI solutions showed a biphasic concentration-dependent cytotoxic response. Indeed, PI at 0.3%, despite being the sole minor most minor toxicity recorded for 3 and 5 min for all cell lines, had toxicity greater than PI at 5% at 1 min for both tenocytes and ligamentous fibroblasts. Our results are comparable to those reported by Goswani et al. [2], who found even lower toxicity of PI at 0.3%. This paradoxical effect has been observed by Gocke et al. [15] and is related to the proportion of free iodine present in the solution. The level of free iodine, the actual active principle of povidone–iodine, depends on the balance between the available amount of iodine and the amount linked to the polymeric complexes forming povidone–iodine. Despite having more iodine molecules functional, high concentration solutions have a higher bound fraction, thus explaining their reduced activity compared with low concentrated ones [16]. Muller et al. [17] evaluated the biocompatibility of povidone–iodine, observing an IC${}_{50}$ of 6.3% on murine fibroblasts after an exposure time of 30 min. Von Kuedel et al. [18] evaluated the toxicity of PI on articular chondrocytes for times of 1, 3, and 6 min with an observed mortality rate higher than ours, with no paradoxical effect reported. However, the study’s significantly different design (histological vs. cytological) and the lines used (human chondrocytes vs. cattle) should be considered. According to our knowledge, no studies in the literature have evaluated the cytotoxicity of different combinations of solutions of PI and H${}_{2}$O${}_{2}$. It has been proven that combining antimicrobials could enhance their activities [9] and help overcome acquired microbial resistance related to the topical use of antibiotics [2,7]. Moreover, unlike other antiseptics, PI and H${}_{2}$O${}_{2}$ do not give rise to deposits and potentially toxic compounds when combined [19]. According to our data, the rank order of cytotoxicity about their toxicity for all cell lines and exposure times was PI at 0.3% < PI at 5% < PI at 0.3% /H${}_{2}$O${}_{2}$ at 0.5% < H${}_{2}$O${}_{2}$ at 0.5% < PI at 5%/ H${}_{2}$O${}_{2}$ at 1.5% < H${}_{2}$O${}_{2}$ at 1.5%. These results show that the combined solutions have lower intermediate toxicity than the individual components at equal concentrations. The most important limitation of the current study is that the results obtained from in vitro experiments cannot necessarily be applied to in vivo findings. Our choice to use cell lines was motivated by the desire to obtain stable long-lasting populations and to avoid a negative effect on the cellular viability of potential therapies and pathological processes of donors [20,21]. However, in vitro cells are not representative of a well-perfused human wound. Human tissue has a higher tolerance for external influences, including antiseptics, than cultured human cells have [17]. Regarding the cell lines chosen, a limitation of this study is the lack of toxicity analysis for bone cell lines in the setting of PJIs. However. this choice was justified by evidence in the literature of the increased risk of embolic phenomena using solutions containing H${}_{2}$O${}_{2}$ into medullary canals [22]. Concerning the tested solutions, we decided not to evaluate the toxicity of solutions containing chlorhexidine, despite its recognized efficacy and widespread use especially in surgery. This choice was dictated by the fact that chlorhexidine can give rise to toxic compounds when used together with other antiseptics [12,19]. Furthermore, using a standard saline solution as diluent excludes the presence of organic compounds that can provide a protective effect against antimicrobial agents; this could lead to underestimating the cytotoxic effects of the antiseptics in our study. Therefore, saline solution lacks a pH buffer, causing weak acidification; this might actually contribute to damage in cells and overestimate toxicity. Howeve, r this effect is very weak and is unlikely to have had a significant result. Another consideration should be made regarding the immune response. Our study did not include cells such as neutrophils and macrophages actively involved in PJIs, but it is good to take into account the possible relationships that the toxicity of these solutions can have on these cells. There is evidence in the literature that this solution exerts some toxicity on these cells [23,24,25], but recent results also seem to suggest that they are being stimulated, with potential effects to promote wound healing [4,26,27]. Further studies will be necessary to verify the toxicity of these solutions on these cell lines.

## 5. Conclusions

Our analysis showed the different behavior of cytotoxicity of PI and H${}_{2}$O${}_{2}$ solutions regarding exposure time and concentration. All solutions tested presented progressive increases in toxicity as exposure time increased, except PI at 0.3%, which exhibited the lowest toxicity. The use of H${}_{2}$O${}_{2}$ 1.5% is not recommended, being the most toxic for all cell lines tested. The combined solutions reported a reduced cellular killing at 3 and 5 min than H${}_{2}$O${}_{2}$ at equal concentrations, while these results were similar to PI solutions. Further studies will be needed to verify the efficacy and in vivo safety of these solutions.

## Figures and Tables

**Table 1:** Antiseptic solutions.

Solutions Tested	Materials ^1^
Hydrogen Peroxide 0.5%	417 mL NaCl 0.9%/83 mL H${}_{2}$O${}_{2}$ 3%
Hydrogen Peroxide 1.5%	250 mL NaCl 0.9%/250 mL H${}_{2}$O${}_{2}$ 3%
Povidone–iodine 0.3%	485 mL NaCl 0.9%/15 mL PI 10%
Povidone–iodine 5%	250 mL NaCl 0.9%/250 mL PI 10%
Povidone–iodine 0.3%/Hydrogen Peroxide 0.5%	15 mL PI 10%/83 mL H${}_{2}$O${}_{2}$ 3%/402 mL NaCl 0.9%
Povidone–iodine 5%/Hydrogen Peroxide 1.5%	250 mL PI 10%/250 mL H${}_{2}$O${}_{2}$ 3%

^1^ Materials shown are intended for a volume of 500 mL.

**Table 2:** Toxicity data in chondrocyte cells.

Tested Solutions	1 min	3 min	5 min	*p* Value
Hydrogen Peroxide 0.5%	10.3 ± 3	31.6 ± 8.1	44.8 ± 10.5	<0.001
Hydrogen Peroxide 1.5%	54.7 ± 1.7	63.7 ± 0.35	74.8 ± 1.6	<0.001
Povidone–iodine 0.3%	18.8 ± 0.53	21.9 ± 0.63	20.7 ± 2.42	0.061
Povidone–iodine 5%	9.33 ± 1.2	31.5 ± 5.54	44.2 ± 4.53	<0.001
Povidone–iodine 0.3%/Hydrogen Peroxide 0.5%	22.4 ± 1	27.9 ± 1.3	29.6 ± 1.35	<0.001
Povidone–iodine 5%/Hydrogen Peroxide 1.5%	16.2 ± 1	31.9 ± 2.4	45.9 ± 3.1	<0.001
*p* value	<0.001	<0.001	<0.001	

The values reported indicate the percentage of dead cells over total cells.

**Table 3:** Toxicity data in tenocyte cells.

Tested Solutions	1 min	3 min	5 min	*p* Value
Hydrogen Peroxide 0.5%	7.2 ± 3.4	23.6 ± 2.3	42.2 ± 1	<0.001
Hydrogen Peroxide 1.5%	46.4 ± 1.6	54.8 ± 3.6	63.3 ± 2	<0.001
Povidone–iodine 0.3%	15.8 ± 1.7	16.9 ± 2.3	18.9 ± 1.7	0.214
Povidone–iodine 5%	7.6 ± 1	20.4 ± 2	29.3 ± 1.8	<0.001
Povidone–iodine 0.3%/Hydrogen Peroxide 0.5%	20.9 ± 1	23.3 ± 2	25.6 ± 1.7	0.034
Povidone–iodine 5%/Hydrogen Peroxide 1.5%	13.6 ± 1.4	26.4 ± 2	36.2 ± 3.1	<0.001
*p* value	<0.001	<0.001	<0.001	

The values reported indicate the percentage of dead cells over total cells.

**Table 4:** Toxicity data in fibroblast-like synoviocytes.

Tested Solutions	1 min	3 min	5 min	*p* Value
Hydrogen Peroxide 0.5%	8.0 ± 0.8	24.7 ± 1.3	45.6 ± 1	<0.001
Hydrogen Peroxide 1.5%	52.7 ± 2.6	54.4 ± 2.5	60.4 ± 4.9	<0.001
Povidone–iodine 0.3%	16.4 ± 0.3	18.9 ± 0.5	20.0 ± 0.7	<0.001
Povidone–iodine 5%	9.8 ± 1	31.3 ± 1.3	38.7 ± 1.3	<0.001
Povidone–iodine 0.3%/Hydrogen Peroxide 0.5%	20.0 ± 1.8	21.8 ± 1.4	25.1 ± 1	0.012
Povidone–iodine 5%/Hydrogen Peroxide 1.5%	14.0 ± 1.3	25.1 ± 1	31.6 ± 1.7	<0.001
*p* value	<0.001	<0.001	<0.001	

The values reported indicate the percentage of dead cells over total cells.

## Data Availability

Datasets collected or analyzed during the current study are available from the corresponding author on request.

## Abbreviations

The following abbreviations are used in this manuscript:

PJIs	Periprosthetic joint infections
PI	Povidone iodine
H${}_{2}$O${}_{2}$	Hydrogen peroxide
HFLS	Human Fibroblast-Like Synoviocytes
NHAC-kn	Normal Human knee Articular Chondrocytes
